# Evaluating Need Crafting: Scale Validation and Workplace Implications

**DOI:** 10.3390/bs14040301

**Published:** 2024-04-05

**Authors:** Ivan Putter, Leoni van der Vaart, Jacqueline Bosman

**Affiliations:** 1 Optentia Research Unit, North-West University, Vanderbijlpark 1900, South Africa; ivan.g.putter@gmail.com (I.P.); jacqueline.bosman@nwu.ac.za (J.B.); 2 Department of Psychology, Norwegian University of Science and Technology, 7491 Trondheim, Norway

**Keywords:** self-determination theory, need crafting, need satisfaction, validity, bifactor modeling, work context, motivational process

## Abstract

Basic psychological need crafting assumes that need-based experiences are enhanced through intentional behavior and thought changes. Despite its known benefits outside of the work context, need crafting instruments designed for this context, and the implications of need crafting for employee functioning, remain underexplored. Thus, this study set out to adapt and validate the need crafting scale (NCS) among employees (*n* = 229). Results supported the construct, discriminant validity, and criterion validity of the NCS. The research also revealed that, through need crafting, employees reported enhanced experiences related to their needs, which led to higher work effort and engagement and a reduced desire to leave their jobs. Additionally, the different types of need crafting had differential direct effects on employee functioning, supporting a more nuanced understanding of the construct. As the first of its kind, the study underpins the relevance and generalizability of the NCS and need crafting in the workplace.

## 1. Introduction

Need crafting involves intentionally altering behaviors or thoughts to fulfil psychological needs [1,2]. From a self-determination theory (SDT) perspective, a person engages in self-directed crafting behavior to satisfy three basic psychological needs: autonomy (i.e., when a person’s actions are experienced as the result of choice), competence (i.e., when a person experiences mastery and efficacy from their interactions with their immediate environment), and relatedness (i.e., when a person experiences a sense of belonging among other people) [3,4,5]. To achieve *autonomy crafting*, one must proactively express one’s feelings, values, interests, and preferences, alongside taking more control over one’s activities and tasks. *Competence crafting* comes about by deliberately creating opportunities for mastery and skill development, driven by a willingness to learn and awareness of one’s capabilities. Lastly, *relatedness crafting* is realized through intentionally building relationships characterized by care, closeness, and sincerity [2]. The desire for active participation in need fulfilment aligns with SDT’s emphasis on people’s proactive nature [2,6].

Despite the infancy of need crafting in SDT, Laporte and colleagues [2] developed and validated a scale for Belgian adolescents to measure this proactive approach to need satisfaction and its positive outcomes. Results supported the psychometric properties of the scale in a non-work context. More specifically, a hierarchical model best represented the scale’s multi-dimensionality, showing moderate to large correlations with the three types of need crafting and substantial correlations with the global need crafting factor. However, they modeled the general need crafting factor using a third-order approach [2]. This approach is rarely implemented and critiqued by methodologists for unrealistic constraints in practice [7,8] and lacking substantive sense [9,10]. Consequently, bifactor models have been favored in which items are simultaneously used to assess their own dimensions and a general factor [10]. Other SDT studies using bifactor modeling successfully demonstrated that psychological need satisfaction [11], motivation [12], and need supportive/thwarting behaviors [13] are best represented by this type of hierarchical model. The current study subsequently used a bifactor model as an alternative to explore the multi-dimensionality of the need crafting scale (NCS) and to source evidence for its construct validity.

The positive outcomes of need crafting were demonstrated through its associations with need-based experiences [2,14], energy, work motivation, and work engagement [15,16]. Intervention studies revealed increased need satisfaction [17], self-regulation, and subjective vitality, along with decreased need frustration, amotivation, perceived stress [18], and depression [19]. These benefits were evident for adolescents [2,14] and adults [17], including those in highly stressful circumstances [18,19]. Another study [20] explored autonomy crafting among employees without adapting the original scale items for the work context. Despite this, the study reported positive outcomes in employee performance linked to autonomy satisfaction and work engagement. These studies highlight need crafting’s significance, but also point to its restricted workplace application. Its underexplored nature in this context limits our understanding of its context-specific relevance and generalizability, potentially resulting in missed opportunities for improving employee functioning and developing effective intervention strategies. Hence, we investigated correlations with proposed ‘outcome’ variables like need satisfaction and the motivational process initiated by need crafting. These associations will also serve as evidence for the criterion validity of the NCS. Furthermore, discriminant validity will be established between need crafting and job crafting to indicate that the two constructs are minimally related [21].

Validating the NCS and exploring the motivational processes initiated by need crafting contribute to the literature in several important ways: First, while SDT traditionally emphasized self-determination [3,22], studies have highlighted others’ rather than one’s own role in achieving it (e.g., [4,23,24]). However, we demonstrate how individuals can proactively meet their own needs, particularly in the workplace, thereby broadening the scope of SDT and its application in occupational psychology. Second, analyzing bifactor models advances the methodological debate within SDT studies [25]. We specifically investigated if need crafting follows a hierarchical structure, similar to basic psychological needs [11], motivation [12], and need supporting/thwarting behaviors [13], allowing for differentiation between the extent of need crafting (a ‘quantitative’ dimension) and the various methods for need crafting (i.e., ‘qualitatively’ different ways of crafting). Third, drawing from and extending the foundational work of Laporte and colleagues [2], our study sheds light on the complex, multi-dimensional nature of need crafting and its operational dynamics (i.e., within and across categories and cross-dimensional) in the workplace. Last, we clarify the conceptual and operational distinctions between these proactive behaviors in differentiating between need and job crafting. On a practical level, we provide organizational leaders with actionable insights for enhancing employee wellbeing, performance, and retention, as well as a psychometrically sound tool for measuring changes in need crafting post-intervention.

## 2. Literature Review and Hypotheses Development

### 2.1. The Bifactor Modeling Framework

Construct-relevant psychometric multi-dimensionality extends to hierarchically ordered constructs in which each item is presumed to reflect a specific and a global (or general) factor [7,26]. For example, each autonomy crafting action item is associated with (a) its specific target factor (i.e., autonomy crafting action) and (b) a general factor (i.e., need crafting). A bifactor model best represents this [7,26,27]. Bifactor models contain a general factor that reflects the shared variance between all scale items and specific factors that reflect the ‘leftover’ variance shared by the items of a specific subscale [28].

People’s need-based experiences are dependent on both contextual circumstances (like other people) and individual characteristics (like awareness and self-knowledge) [5]. When people are aware of circumstances or people that are need-conducive to them, they become more motivated to engage in need crafting activities proactively [29]. While need crafting acknowledges the importance of awareness, it also emphasizes the corresponding action. Once aware, a person should actively engage and act on it, pursuing identified need-satisfying elements. Therefore, each need crafting factor in the NCS has awareness and action items, resulting in six factors, autonomy awareness, autonomy action, competence awareness, competence action, relatedness awareness, and relatedness action crafting at the first-order level of the hierarchical model [2].

Previous studies support the idea that the different types of need crafting are interrelated. At the second-order level of the hierarchical model, studies have found meaningful correlations between autonomy and relatedness crafting (*r* = 0.44), autonomy and competence crafting (*r* = 0.56), and relatedness and competence crafting (*r* = 0.35). All of these were also highly related to a general need crafting factor at the third-order level: autonomy (*r* = 0.84), relatedness (*r* = 0.77), and competence (*r* = 0.78) [2]. The correlations between the specific need crafting factors together with their associations with the general need crafting factor may be indicative of a hierarchical factor structure. Consequently, the different types of need crafting are expected to be theoretically related. Therefore, we hypothesized:

**Hypothesis** **1** **(H1):** *The NCS’s multidimensionality is best represented by a bifactor solution.*

### 2.2. Discriminant Validity

Discriminant validity could be established through associations between the need and job crafting dimensions, as the latter is presumed to be closely related to the former. Indeed, need crafting stems from job crafting [2] and shares proactivity. However, whereas job crafting aims to create more meaning [30] or fit between the individual and their job’s characteristics (as explained by the job-demands resources model) [31], need crafting aims to enhance need satisfaction [2], in line with SDT. Consequently, it was expected that:

**Hypothesis** **2** **(H2):** *The NCS would display sufficient discriminant validity.*

### 2.3. Criterion-Related Validity and the Motivational Process

Need satisfaction focuses on a person’s basic psychological needs and the levels of satisfaction they experience in relation to their autonomy, competence, and relatedness needs [32,33]. Need satisfaction is both the goal (i.e., to satisfy psychological needs) and the consequence (i.e., satisfied needs) of need crafting endeavors [1]. Need satisfaction transpires once people engage in need crafting behavior [2,14]. It is thus hypothesized that:

**Hypothesis** **3** **(H3):** *Need crafting will associate positively with need satisfaction.*

As a spin-off, need satisfaction relates positively to behaviors, cognitions, and wellbeing [34,35,36]. An internalization process could explain this spin-off, during which need fulfilment leads to individuals engaging in activities for internal rather than external reasons [5,20]. Because the reasons for behaviors are internalized, individuals experience higher levels of wellbeing and performance [3,5].

Work engagement is a wellbeing indicator and is seen as a person’s mental dedication and energy levels at work, together with their sense of challenge, concentration, significance, inspiration, and pride in their work [37]. Work engagement is operationalized by *vigor* (characterized as a person with high energy levels and mental resilience, who is prepared to exert effort and display perseverance in their work, even during difficulty), *dedication* (“feelings of a sense of significance, enthusiasm, inspiration, pride, and challenge”) [37] (pp. 74–75), and *absorption* (characterized by a person who is deeply captivated in their work, with such concentration that time goes by quickly and they struggle to detach themselves from their work) [37,38]. Feeling autonomous, capable, and socially connected (because of need crafting) not only internalizes behavior but is the psychological resource fueling mental dedication and energy levels (vigor), sense of challenge and significance (dedication), and deep concentration (absorption) at work. It is thus hypothesized that:

**Hypothesis** **4** **(H4):** *Need crafting will indirectly affect work engagement positively through need satisfaction.*

Work effort is a behavioral indicator that measures a person’s persistence, direction, intensity, and degree of motivation towards applying effort in their job [39]. Through the process of internalizing behavior, which stems from need crafting and satisfaction, employees are empowered to select goal-oriented behaviors (direction), commit to these goals with vigor (intensity), and maintain their efforts (persistence). It is thus hypothesized that:

**Hypothesis** **5** **(H5):** *Need crafting will indirectly and positively affect work effort through need satisfaction. Turnover intention is a cognitive indicator that looks at an employee’s intention to leave their current employer before indeed leaving [40]. Designing jobs that align with an individual’s needs can lead to inherently rewarding roles and positive workplace relationships. This alignment can make employees more content with their current situation, reducing their desire to leave due to dissatisfaction with their job or work environment. Consequently, it is hypothesized that:*

**Hypothesis** **6** **(H6):** *Need crafting will indirectly affect turnover intention negatively through need satisfaction. These indirect associations would also be indicative of the NCS’s criterion validity.*

While our focus was on the motivational process sparked by need crafting and its indirect effects, it is plausible that roles offering inherent rewards—marked by autonomy and development opportunities—along with positive workplace interactions, can directly boost employee engagement, encourage greater effort, and diminish the desire to leave the organization. Therefore, it is hypothesized that:

**Hypothesis** **7** **(H7):** *Need crafting will directly affect work engagement.*

**Hypothesis** **8** **(H8):** *Need crafting will directly affect work effort.*

**Hypothesis** **9** **(H9):** *Need crafting will directly affect turnover intention.*

## 3. Materials and Methods

### 3.1. Participants and Procedure

A cross-sectional design was used to administer the NCS to white-collar employees of a South African earth-drilling and rock-boring organization, using criteria and snowball sampling methods. Only white-collar employees were included due to the potential risks associated with crafting among machine operators in the mining sector [41]. Participants were recruited via word-of-mouth (i.e., gatekeepers and departmental meetings), flyers, and internal mailing lists. Prospective participants were informed of the study, its voluntary and confidential nature, and its independence from the organization. From a population of 612 employees meeting specific criteria (i.e., South African-based employee, aged 18 to 65, employed by the organization for at least one month, English proficiency, minimum Grade 12 qualification, and not a machine operator), a final sample of 299 employees was obtained (48.80% of the population). Most were male (76.30%) (as is typical in this sector), and about half had a Grade 12 qualification (49.80%). The mean age was 36.2 years (SD = 10.50), with nearly 70% of the sample aged under 40. The sample comprised 42.80% African and 51.50% white employees. Remote working was infrequent, with 71.60% never working from home. The mean organizational tenure of participants was just over six years (SD = 6.35), and the mean position tenure was almost five years (SD = 5.01).

Participants were asked to identify their department within the organization, leading to two main categorizations: functional support and operational staff. Functional support staff included departments such as human resources, finances, engineering, commercial, information technology, and safety, health, environment, and quality. The operational category comprised general workers, workshop and boiler shop artisans, production, operations, site workers, etc. Of the sample, 34% were functional support staff, while 66% were operational staff.

### 3.2. Measuring Instruments

An adapted version of the short form of the *Need Crafting Scale* (NCS) [2] was administered to measure the extent to which participants crafted. To adapt the instrument for the work context, we made minor changes to the instructions and item wording to better suit the working context: references to experiences or activities in ‘their lives’ were changed to ‘at work’ or ‘in the work environment’ and ‘people’ were replaced with ‘colleagues’. After that, it was sent to two subject matter experts who independently commented on the changes before it was distributed to participants. We opted for the shortened version (created by Laporte et al. [2] based on the psychometric properties of items in the extended version) to minimize participant fatigue and maintain engagement. The shortened version comprised 21 items (with seven items per need) rated on a 5-point Likert scale ranging from 1 (*completely not true*/*disagree*) to 5 (*completely true*/*agree*). The questionnaire explored two aspects of need crafting for each of the three needs: awareness and action. Examples of items included “*I know well which activities I am good at*” (competence crafting awareness), “*I deliberately choose activities I am good at*” (competence crafting action), “*I know well which activities I really want to do*” (autonomy crafting awareness), “*I deliberately choose to engage in activities I really want to do”* (autonomy crafting action), “*I know well which colleagues really care about me*” (relatedness crafting awareness), and “*Even when I feel lonely, I still try to contact colleagues who care for me*” (relatedness crafting action). Participants familiarized themselves with the needs first (based on descriptions from [42]) to facilitate understanding, after which they were asked to provide practical examples of activities that satisfied those workplace needs. They then completed the questionnaire about the listed activities [2].

The *Job Crafting Questionnaire* (JCQ) [43], later adapted to a 9-item questionnaire by [44], measured participants’ cognitive, task, and behavioral crafting. The questionnaire was rated on a 5-point Likert scale ranging from 1 (*never*) to 5 (*very often*). Examples of each type of crafting included the following: “*Think about how your job gives your life purpose*” (cognitive crafting), “*Introduce new approaches to improve your work*” (task crafting), and “*Engage in networking activities to establish more relationships*” (relational crafting) [44].

The *Basic Psychological Need Satisfaction and Frustration Scale—Work Domain* (BPNSFS-WD) [32,33] measured participants’ basic psychological need satisfaction. We used only the need satisfaction items, which amounted to 12 items, four for each of the three needs. The three subscales used measured autonomy satisfaction (e.g., “*At work, I feel that I do what really interests me.*”), competence satisfaction (e.g., “*I feel capable in doing what I do at work.*”), and relatedness satisfaction (e.g., “*I experience a warm and good feeling with the people I spend time with at work.*”). A 7-point Likert-type scale captured participant’s responses, ranging from 1 (*strongly disagree*) to 7 (*completely agree*) [32,33].

The *Utrecht Work Engagement Scale (UWES-9)* captured participants’ work engagement with nine items, three for each of the dimensions (i.e., vigor, dedication, and absorption) [38]. Each self-reported item was completed on a 7-point frequency scale, ranging from 0 (*never*) to 6 (*always*). Three example items were “*At work, I feel bursting with energy*” (vigor), “*I am enthusiastic about my job*” (dedication), and “*I am immersed in my work*” (absorption) [38].

The *Work Effort Scale (WESC)* measured participants’ persistence, direction, and intensity on a 7-point Likert-type scale, ranging from 1 (*fully disagree*) to 7 (*fully agree*). Examples included “*When I start an assignment, I pursue it to the end*” (persistence), “*I really do my best to achieve the objectives of the organization*” (direction), and “*I really do my best in my job*” (intensity) [39].

The Turnover Intention Scale [45] consisted of only three items which looked at overall turnover propensity: “I am actively looking for other jobs”, “I feel that I could leave this job”, and “If I was completely free to choose, I would leave this job”. This instrument was measured on a 5-point Likert scale, ranging from 1 (strongly disagree) to 5 (strongly agree) [45].

### 3.3. Data Analyses

Mplus 8.10 [46] was used for latent variable analysis (LVA). Furthermore, jamovi (version 2.3) [47] was used for descriptive statistics. LVA was used to determine the different instruments’ factor structures (i.e., confirmatory factor analysis [CFA]), using the weighted least square mean and variance adjusted (WLSMV) estimator. The WLSMV estimator is preferred when using categorical data [48]. The omega (ω) coefficient, as introduced by [49], is highlighted for its flexibility, straightforward calculation, and direct link to the characteristics of the chosen measurement model [50]. Consequently, we used this method to estimate the composite reliabilities.

The following absolute and comparative fit indices were used to determine the fit between the data and the measurement models: chi-square (χ^2^), degrees of freedom (d*f*), root mean square error of approximation (RMSEA), standardized root mean square residual (SRMR), the comparative fit index (CFI), and the Tucker–Lewis index (TLI). Even if the chi-square is significant, the model should not be discarded, as the chi-square is often strongly influenced by sample size [48]. When the values of CFI and TLI exceed 0.95 [51], they are seen as indicators of a good fit, but values of 0.90 and higher are also acceptable. RMSEA values lower than 0.08 and SRMR values lower than 0.10 indicate a proper fit [48]. The effect sizes for correlations were interpreted on the following cut-off scores: *r* ≥ 0.10 (small effect), *r* ≥ 0.30 = moderate, and *r* ≥ 0.50 = large [52].

Mediation analyses were executed to determine both the direct and indirect effects of need crafting. The mediation procedure, which entails bootstrapping with a minimum of 5000 samples, was used. Through bootstrapping, 95% confidence intervals were generated, and when the upper-level confidence intervals (ULCI) and lower-level confidence intervals (LLCI) did not include zero, a significant indirect effect existed [53]. The factor scores from the measurement model containing the need crafting and ‘outcome’ variables were exported for the mediation analysis. For the mediation analysis, we used PROCESS macro for Model 4 [53] in RStudio [54].

## 4. Results

Table 1 indicates the competing measurement models tested to determine the best-fitting model. Model 1a included the six crafting factors (i.e., autonomy, competence, and relatedness awareness; and autonomy, competence, and relatedness action). In this model, the items were regressed onto their a priori factors and the six factors were allowed to correlate. Model 2 was similar, but an extra factor, a method factor, was added as the last item of each subscale, which was negatively phrased [2]. While differing in content from their positive counterparts, these negatively worded items may share a common (i.e., negative) method. Negatively phrased questions tend to cluster together, irrespective of their subscales, leading to the introduction of a method factor to account for the variance shared among them [55].

**Figure 1:**
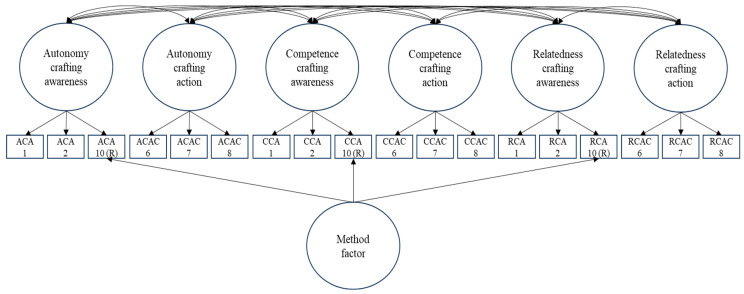
Simplified representation of Model 2.

After specifying Model 2, the three negatively phrased action items were deleted as they did not load significantly onto their a priori factors when controlling for the method factor. Figure 1 visually represents this model. We also deleted these items from the previous model for comparison purposes, resulting in Model 1b. We retained each awareness component’s negatively phrased item since they loaded significantly onto their a priori factors and the method factor.

Models 3 and 4 both represented hierarchical models. In Model 3, the items were allowed to load onto their a priori (also called specific) factors and onto a general factor that captures need crafting. All factors in this model were orthogonal, as [27] recommended. In Model 4, the items were allowed to load onto their a priori factors, and these lower-order factors were then regressed onto a higher-order (or second-order) need crafting factor. The lower-order factors were uncorrelated with each other, as recommended by [27]. Figure S1 in the Supplementary Materials visually represent Models 1b, 3 and 4.

Table 1 shows that the correlated six-factor model, with a method factor for the negatively phrased items, delivered the best fit, highest CFI and TLI, and the lowest RMSEA and SRMR values. These fit indices also met their pre-determined cut-off criteria. The findings also revealed that the general factor was poorly defined, evidenced by small factor loadings on the general factor, particularly in comparison to the loadings on the specific factors. Therefore, Hypothesis 1 is unsupported. All subsequent analyses were based on Model 2.

In addition to model fit, the models’ factor loadings were also evaluated. Items’ standardized factor loadings should ideally be above 0.70, with loadings higher than 0.50 also being acceptable [56]. As can be seen from Table 2, all factor loadings were significant, and most were above 0.50.

The correlations between the six need crafting factors are presented in Table 3. All correlations were significant, in the expected direction, and mostly had medium to large effect sizes. Within the same category, the awareness components correlated very strongly (*r* = 0.52 to 0.76), particularly between autonomy and competence awareness (*r* = 0.76). These associations indicate a significant interplay in participants’ cognitive perceptions across these domains. At the same time, the action components also showed strong correlations among themselves (*r* = 0.49 to 0.61), notably between competence and related action (*r* = 0.61). These correlations suggest that behaviors aimed at meeting these needs are interconnected. The cross-category correlations (awareness to action for the same need) were large (*r* = 0.51 to 0.70), particularly between relatedness awareness and action (*r* = 0.70). These correlations indicate that an individual’s awareness of how a particular need could be satisfied strongly links with their actions to fulfill that need. While the correlations between awareness in one domain and actions in another (i.e., cross-dimensional correlations) are present (*r* = 0.22 to 0.67), they are generally weaker than those in the same category or need. This indicates that awareness of how one need (e.g., competence) could be satisfied is associated with the actions aimed at fulfilling a different need (e.g., autonomy or relatedness). The reliability coefficients exceeded 0.70, except autonomy action crafting (ω = 0.57). Together, the correlations and internal consistency coefficients further support the instruments’ factorial structure.

The above model fit, factor loadings, reliability, and correlations within the NCS support the scale’s construct validity.

### 4.1. Discriminant Validity

Discriminant validity was evaluated by assessing the correlations between the JCQ and the NCS. Correlations between the two instruments should not exceed 0.80, which could indicate that the two instruments essentially measure the same construct [57]. The significant correlations were mostly small-to-medium in effect size (*r* = 0.15 to 0.48), indicating the two constructs’ relatedness yet independence (see Table 4).

As seen in Table 4, the need crafting factors mostly had medium to large (i.e., strong) correlations among themselves (*r* = 0.23 to 0.76). The only exception was the correlation between autonomy action and relatedness awareness (*r* = 0.23), which is a small effect. Similarly, strong correlations were observed between the job crafting dimensions (*r* = 0.23 to 0.74). The correlation between relatedness and cognitive crafting was especially strong (*r* = 0.74), suggesting a tight interplay between how individuals shape their social connections and their mental strategies for engaging with tasks. The correlations between need crafting and job crafting show that only small-to-medium effect sizes existed (*r* = 0.12 to 0.48), indicating discriminant validity between the two instruments. These findings were, therefore, supportive of Hypothesis 2. Interestingly, cognitive crafting shows a stronger relationship with the need crafting types than task and relatedness crafting.

### 4.2. Criterion Validity

Criterion validity was assessed by incorporating need satisfaction, turnover intention, and work effort into the structural model. As depicted in Table 5, the relationships between the need crafting factors and the ‘outcome’ variables were significant (except between competence awareness and turnover intention) and in the expected directions. Need crafting was positively related to need satisfaction (supporting Hypothesis 3), engagement, and work effort. Need crafting was negatively associated with turnover intention. Furthermore, there were significant correlations between the need crafting elements and their relevant need satisfaction elements. For example, autonomy action and autonomy awareness were strongly associated with autonomy satisfaction (*r* = 0.56 and *r* = 0.50). Similarly, this was the case with competence action and competence awareness in relation to competence satisfaction (*r* = 0.66 and *r* = 0.55), as well as with relatedness action and relatedness awareness in relation to relatedness satisfaction (*r* = 0.57 and *r* = 0.50).

Mediation analysis was conducted to test the hypotheses of the need crafting dimensions’ indirect effect on the identified ‘outcome’ variables (i.e., work engagement, turnover intention, and work effort). The motive behind each crafting component is to enhance its associated need-based experience. For example, autonomy crafting is aimed towards enhancing autonomy satisfaction. Consequently, 18 different models were specified; six crafting dimensions multiplied by three outcomes. Although the crafting awareness and action dimensions were modeled separately, we controlled for the opposite dimension in each model. For example, when we modeled the outcomes of autonomy crafting awareness, we controlled for the effect of autonomy crafting action by adding it as a covariate in the model. The results of the indirect effects are reported in Table 6, and the direct effects in Table 7. The supplementary file shows the indirect effect figures (Figures S2–S19) to save space.

Table 6 shows that autonomy crafting awareness had positive indirect associations with work engagement (β = 0.42) and work effort (β = 0.06), whereas it had a negative indirect association with turnover intention (β = −0.34). Table 7 shows that the direct effects of autonomy crafting awareness on work effort (β = 0.48) and turnover intention (β = 0.29) were significant, but this was not the case for work engagement (β = 0.03). Therefore, it could be argued that autonomy crafting awareness only indirectly affects work engagement. The direct effect on turnover intention was positive rather than negative, contradicting expectations. Autonomy crafting action also had positive indirect associations with work engagement (β = 0.41) and work effort (β = 0.06) but a negative indirect association with turnover intention (β = −0.33). The direct effects of autonomy crafting action were only significant for work engagement (β = −0.12). This negative effect contradicts expectations. Resultantly, autonomy crafting action directly and indirectly affected work engagement, whereas it only indirectly affected work effort and turnover intention.

Competence crafting awareness had positive indirect associations with work engagement (β = 0.54) and work effort (β = 0.27) but a negative indirect association with turnover intention (β = −0.26). The direct associations of competence crafting awareness, in turn, were significant for all three factors: work engagement (β = −0.21), turnover intention (β = 0.49), and work effort (β = 0.29). These negative and positive effects on work engagement and turnover intention contradict expectations. Nevertheless, all three factors (i.e., work engagement, turnover intention, and work effort) had significant indirect and direct associations with competence crafting awareness. Competence crafting action had positive indirect associations with both work engagement (β = 0.28) and work effort (β = 0.14) but a negative indirect association with turnover intention (β = −0.13). However, the direct effect of competence crafting action was only significant for turnover intention (β = −0.36). Competence crafting action had indirect and direct associations with turnover intention but only indirect associations with work engagement and work effort.

Relatedness crafting awareness had positive indirect associations with work engagement (β = 0.31) and work effort (β = 0.13) but a negative indirect association with turnover intention (β = −0.25). The direct associations with relatedness crafting awareness were significant for both work engagement (β = −0.19) and work effort (β = 0.45) but not for turnover intention (β = 0.01). This negative effect contradicts expectations. Relatedness crafting action also had positive indirect associations with both work engagement (β = 0.10) and work effort (β = 0.04) but a negative indirect association with turnover intention (β = −0.25). The direct effects from relatedness crafting action were only significant with work engagement (β = 0.25). Relatedness crafting action, directly and indirectly, affected work engagement, whereas it only indirectly affected work effort and turnover intention.

In conclusion, the results provided support for Hypotheses 4 to 6. In supporting these hypotheses, additional evidence is provided for the criterion validity of the NCS. Hypotheses 7 to 9 received partial support because not all the direct relationships examined were significant.

## 5. Discussion

The study aimed to validate an adapted version of the short form of the NCS within a work context using a bifactor framework. Additionally, it aimed to gather evidence for the instrument’s discriminant and criterion validity and to explore the motivational process through which need crafting associates with employee functioning.

Our results supported a six-dimensional structure where the dimensions of need crafting were strongly related. Contrary to the hypothesis and previous findings [2], a single overarching factor explaining their commonality was absent. Consequently, we conclude that the need crafting dimensions are connected (and therefore ‘qualitatively’ distinct), but they are not uniformly influenced by a single (or ‘quantitative’) need crafting factor. Put differently, employees had distinct perceptions of their specific need crafting activities but lacked a general impression of need crafting as a whole. This also indicates that no total overall score should be considered for the NCS. This outcome is not surprising when considering that there is neither an underlying continuum of need crafting, nor any predictable ordering between the different types of need crafting, unlike the motivational regulations outlined in SDT. We suggest that researchers aim to duplicate these results, even if only employing bifactor modeling for methodological reasons (e.g., capturing construct-relevant multi-dimensionality), similar to our approach. The items were found to be valid indicators of their a priori specified factors and mostly measured them consistently. Our results also yielded three interesting insights into the internal dynamics of the NCS. First, the correlation coefficients indicated a strong interconnectedness within the awareness and action components, underscoring a coherent perception of how needs can be crafted and a unified approach to crafting them. Second, the translation from awareness to action is strong but varies by need, with relatedness showing a stronger awareness–action link compared to autonomy and competence. Third, cross-dimensional interpretations revealed that awareness in one domain (especially autonomy) significantly associates with actions related to other needs. Together, these results provide evidence for the construct validity of this adapted version of the NCS.

The instrument yielded sufficient discriminant validity with job crafting, indicating it is related to but different from job crafting despite originating from it [2]. Furthermore, results supported the criterion validity of the NCS as the need crafting dimensions related positively to need satisfaction, work effort, work engagement, and turnover intention. These results support earlier findings demonstrating that need crafting initiatives benefit need-based experiences, wellbeing, and performance [2,14,17,20].

Moreover, need crafting initiated a motivational process that positively affected work engagement and effort while negatively impacting turnover intention. These findings mean that employees who engaged in need crafting felt more involved, enthusiastic, and committed to their work. They also put more effort into their job tasks. At the same time, these employees were less likely to consider leaving their jobs. This aligns with the research of [14], which discovered that adolescents’ wellbeing is directly impacted by need crafting and also mediated by need satisfaction. In SDT research, need satisfaction often serves as a mediator to explain the motivational processes affecting employee performance and wellbeing [20,58]. The current study corroborates these earlier findings and further supports the criterion validity of the NCS.

Some notable direct associations support the importance of need crafting in achieving its aim of improving need-based experiences. For example, while employees’ awareness of opportunities to craft autonomy, competence, and relatedness positively influenced their work effort, an increased awareness of their potential to craft for autonomy and competence was associated with a higher intention to leave the organization. Consequently, fulfilling these needs becomes crucial in counteracting the effect of mere awareness on turnover intentions. Similarly, when employees took control of their tasks and recognized their strengths and supportive colleagues, they experienced reduced work engagement (in the absence of need fulfilment). These direct, unintended consequences are consistent with cognitive dissonance theory [59], suggesting that people may experience negative outcomes when there are discrepancies in their mental experiences, such as engaging in crafting that does not lead to positive need-based experiences.

It is noteworthy that relatedness crafting generally poses fewer risks in terms of direct unintended consequences. Aside from the case where awareness of potential positive interactions reduced work engagement without relatedness satisfaction, relatedness crafting showed more positive than negative outcomes. For instance, employees who recognized the value of good relationships within the organization not only invested more effort and were less inclined to leave but also became more engaged when they actively created or engaged in supportive relationships. The link with reduced intentions to leave aligns with job embeddedness theory [60]—strong formal or informal connections in the organization reduce the likelihood of leaving due to reluctance to lose these connections [61]. Furthermore, actively pursuing positive interactions underscores the engaging nature of such relationships. We recommend additional research into identifying which specific dimensions of crafting trigger motivation, and what types of motivation are elicited, to gain deeper insights into its outcomes. Previous studies support this (refer to [62] for a comprehensive review), which have shown that the quality of motivation plays a significant role in explaining employee functioning. Another fruitful avenue is to include individual differences in motivational orientations (i.e., autonomy, controlled, and impersonal) that could moderate the relations.

### Theoretical and Practical Implications of This Study

The current study advances our understanding of need crafting in multiple ways. It provides evidence for the psychometric properties of the adapted NCS in a work setting. Contradicting prior research [2], our findings suggest that the scale is not best represented by a singular overarching factor, whether modeled as a second-order or a bifactor. This finding highlights the complex nature of need crafting and suggests that it should not be reduced to a single dimension. Furthermore, it reveals that not all constructs within SDT are suited to hierarchical organization. Extending the work of [2], our work highlights the internal dynamics of the NCS; awareness and action are interconnected with stronger relationships within the same category or need, and with more nuanced relationships across different dimensions. We also established that need crafting is distinct from job crafting despite its origins in the latter. This distinction underscores our argument that, despite their shared proactive basis, need crafting and job crafting are conceptually and operationally distinct. This distinction (together with the findings on employee functioning) is also crucial for understanding the role need crafting plays in employee behavior and wellbeing.

Regarding employee functioning, the study showed that need crafting successfully improved need-based experiences, leading to positive outcomes like increased work effort and engagement and decreased intention to leave. In this way, we broaden SDT by acknowledging the proactive role of the individual in shaping their need-based experiences and work-related functioning. However, removing need satisfaction from the equation resulted in varying effects based on the type of crafting (and action vs. awareness) and the specific outcome, suggesting a need for a more nuanced approach to the construct. These unique outcomes further verify that each form of need crafting is uniquely different, reinforcing the argument that they should not be merged into a single need crafting score. This stance aligns with [34], who made a similar case regarding the basic psychological needs. The differential impact of awareness versus crafting raises the question of whether awareness should be considered an integral part of need crafting, a topic we revisit later. Overall, the study underscores the relevance and generalizability of the NCS and need crafting in the workplace.

## 6. Limitations and Recommendations for Future Research

A few limitations to the study are worth mentioning and should be considered when results are interpreted. First, the study relied on cross-sectional data. While cross-sectional studies may be valuable for investigating new areas [63], as is the case with need crafting, they have limitations in establishing causality and calculating mediating effects. Their specified time frames can also introduce memory biases [64,65]. Second, the study was conducted in a single organization with a relatively small sample. While this could affect the generalizability of the results, [21] mention that validation studies could be conducted incrementally, starting with a single organization and extending to nationwide or even cross-cultural studies. Third, while self-reporting still holds various noteworthy advantages [66], it may lead to common method variance [67] and differences between cultures [68]. Fourth, despite using various strategies to ensure a heterogenous sample, potential selection bias could affect the findings [69]. Fifth, the reliability of the autonomy crafting action scale continues to be an issue. Other researchers have previously noted this problem and attributed it to the scale’s lack of adaptation in the workplace [20]. Despite our efforts to adapt the scale in this study, the issue persisted. Future research should focus on using (intensive) longitudinal designs among larger and more representative samples. Last, given that the negatively phrased action items did not significantly load onto their pre-determined factors after accounting for the method factor, we advise researchers to keep these items in their initial models pending further evidence in other contexts.

Generally, the variance in outcomes between awareness and action components is not surprising, as these components often yield different results [70,71]. However, future research could help better understand the differential direct associations depending on whether it is awareness or action and the outcomes at play. Furthermore, individuals must be aware of resources that can satisfy their needs. Once aware, they can then take steps to meet those needs [2]. However, while awareness is crucial for initiating the crafting process, it may not be a direct part of the crafting activity. Including awareness as part of the process may lead to confusion between causes and effects. Moreover, awareness might coincide with or come before a person’s confidence in their crafting abilities, a key factor for acting [72]. Thus, we suggest that future studies explore how awareness and action related to need crafting occur in sequence rather than simultaneously. The field could benefit from incorporating additional sources of need satisfaction, such as supervisors’ interpersonal styles [73], to provide a more comprehensive understanding from both top-down and bottom-up perspectives. While the current study focused solely on need satisfaction, future research should examine the roles of need unfulfillment and frustration [74] as potential mediators.

## 7. Conclusions

Our study has made significant strides in understanding need crafting within the workplace, revealing a nuanced six-dimensional structure of the construct. This structure, where dimensions of need crafting are closely interrelated yet distinct, along with the high validity of items for their specified factors, underscores the construct validity of the adapted NCS. Additionally, the NCS demonstrated sufficient discriminant validity from job crafting, affirming that, although related and sharing a common origin (i.e., proactivity), need crafting represents a distinct concept. The results further bolster the criterion validity of the NCS, showing positive correlations with need satisfaction, work effort, and work engagement, and a negative correlation with turnover intention. This indicates that need crafting not only differentiates from job crafting but also actively enhances employees’ work experience and inclination to stay within an organization. Engaging in need crafting activities seems to initiate a motivational process, leading to increased work engagement and effort and a decreased desire to leave.

However, the study also points to the complexity of need crafting’s impact when need satisfaction is excluded, highlighting that the effects vary depending on the specific type of crafting (action vs. awareness) and the outcome considered.

## Figures and Tables

**Table 1:** Measurement models (n = 299).

Model	χ^2^	d*f*	RMSEA [90% CI]	CFI	TLI	SRMR
1a. CFA: 6 factors	1084.731 *	174	0.132 * [0.132, 0.140]	0.816	0.777	0.096
1b. CFA: 6 factors	560.526 *	120	0.111 * [0.112, 0.120]	0.902	0.875	0.070
2. CFA: 6 factors + MF	395.348 *	117	0.089 * [0.080, 0.099]	0.938	0.919	0.060
3. Bifactor-CFA + MF	419.134 *	114	0.095 * [0.085, 0.104]	0.932	0.909	0.066
4. H-CFA + MF	469.152 *	126	0.095 * [0.086, 0.105]	0.924	0.907	0.071

Notes. CFA—confirmatory factor analysis; H-CFA—hierarchical (second-order) confirmatory factor analysis; MF—method factor; χ^2^—chi-square; d*f*—degrees of freedom; RMSEA—root mean square error of approximation; CFI—comparative fit index; TLI—Tucker–Lewis index; CI—confidence intervals; SRMR—standardized root mean squared residual; * *p* <0.001.

**Table 2:** Standardized factor loadings.

	Estimate	S.E.
Competence crafting awareness BY		
CCA_1	0.894	0.030
CCA_2	0.906	0.028
CCA_10	−0.530	0.053
Competence crafting action BY		
CCAC_6	0.876	0.040
CCAC_7	0.598	0.053
CCAC_8	0.454	0.050
Autonomy crating awareness BY		
ACA_1	0.880	0.031
ACA_2	0.854	0.029
ACA_10	−0.385	0.053
Autonomy crating action BY		
ACAC_6	0.280	0.059
ACAC_7	0.831	0.068
ACAC_8	0.504	0.057
Relatedness crafting awareness BY		
RCA_1	0.904	0.026
RCA_2	0.869	0.029
RCA_10	−0.516	0.049
Relatedness crafting action BY		
RCAC_6	0.773	0.034
RCAC_7	0.676	0.036
RCAC_8	0.884	0.032
Negative method BY		
CCA_10	0.692	0.064
ACA_10	0.599	0.057
RCA_10	0.401	0.049

Notes. SE—standard error; CCA—competence crafting awareness; CCACT—competence crafting action; ACA—autonomy crafting awareness; ACAC—autonomy crafting action; RCA—relatedness crafting awareness; RCAC—relatedness crafting action. All *p*-values were ≤0.001.

**Table 3:** Descriptive statistics, internal consistency coefficients, and correlations.

	Mean	SD	1	2	3	4	5	6
Competence awareness	40.39	0.60	0.90					
2. Competence action	40.11	0.69	0.58 ***	0.69				
3. Autonomy awareness	40.29	0.61	0.76 ***	0.67 ***	0.82			
4. Autonomy action	30.44	0.82	0.29 ***	0.54 ***	0.51 ***	0.57		
5. Relatedness awareness	40.13	0.75	0.52 ***	0.54 ***	0.61 ***	0.22 ***	0.84	
6. Relatedness action	30.93	0.87	0.45 ***	0.61 ***	0.52 ***	0.49 ***	0.70 ***	0.82

Notes. SD—standard deviation; *** *p* ≤ 0.001. Omega coefficients are reported on the diagonal.

**Table 4:** Internal consistency coefficients and correlations between need and job crafting.

	1	2	3	4	5	6	7	8	9
Competence awareness	0.90								
2. Competence action	0.59 ***	0.69							
3. Autonomy awareness	0.76 ***	0.67 ***	0.82						
4. Autonomy action	0.29 ***	0.53 ***	0.51 ***	0.57					
5. Relatedness awareness	0.52 ***	0.54 ***	0.61 ***	0.23 ***	0.84				
6. Relatedness action	0.45 ***	0.61 ***	0.52 ***	0.48 ***	0.70 ***	0.82			
7. Task crafting	0.15 *	0.37 ***	0.26 ***	0.33 ***	0.12	0.19 **	0.62		
8. Relatedness crafting	0.25 ***	0.17 **	0.26 ***	0.32 ***	0.26 ***	0.29 ***	0.23 **	0.80	
9. Cognitive crafting	0.43 ***	0.42 ***	0.48 ***	0.38 ***	0.37 ***	0.41 ***	0.34 ***	0.74 ***	0.85

Notes. *** *p* ≤ 0.001; ** *p* ≤ 0.01; and * *p* ≤ 0.05. Omega coefficients are reported on the diagonal.

**Table 5:** Internal consistency coefficients and correlations between need crafting and the ‘outcome’ variables.

	1	2	3	4	5	6	7	8	9	10	11	12
1. Competence awareness	0.90											
2. Competence action	0.60 ***	0.67										
3. Autonomy awareness	0.76 ***	0.68 ***	0.82									
4. Autonomy action	0.27 ***	0.44 ***	0.47 ***	0.58								
5. Relatedness awareness	0.51 ***	0.54 ***	0.61 ***	0.24 ***	0.85							
6. Relatedness action	0.44 ***	0.62 ***	0.52 ***	0.45 ***	0.70 ***	0.82						
7. Autonomy satisfaction	0.29 ***	0.48 ***	0.56 ***	0.50 ***	0.28 ***	0.44 ***	0.75					
8. Competence satisfaction	0.66 ***	0.55 ***	0.69 ***	0.37 ***	0.44 ***	0.44 ***	0.72 ***	0.86				
9. Relatedness satisfaction	0.36 ***	0.43 ***	0.46 ***	0.33 ***	0.57 ***	0.50 ***	0.65 ***	0.58 ***	0.89			
10. Work engagement	0.40 ***	0.35 ***	0.46 ***	0.35 ***	0.29 ***	0.33 ***	0.80 ***	0.65 ***	0.47 ***	0.88		
11. Work effort	0.54 ***	0.44 ***	0.50 ***	0.37 ***	0.45 ***	0.34 ***	0.40 ***	0.59 ***	0.39 ***	0.55 ***	0.95	
12. Turnover intention	−0.05	−0.22 ***	−0.14 *	−0.23 ***	−0.19 **	−0.14 *	−0.55 ***	−0.24 ***	−0.35 ***	−0.61 ***	−0.25 ***	0.91

Notes. *** *p* ≤ 0.001; ** *p* ≤ 0.01; and * *p* ≤ 0.05. Omega coefficients are reported on the diagonal.

**Table 6:** Indirect effect of need crafting on employee ‘outcomes’ via need satisfaction.

Indirect Effect	Estimate	BootSE	Boot95%CI
**Autonomy crafting awareness**			
ACA → AUTSAT → ENGAGE	0.42	0.05	[0.32, 0.53]
ACA → AUTSAT → TURN	−0.34	0.05	[−0.45, −0.24]
ACA → AUTSAT → EFFORT	0.06	0.03	[0.00, 0.13]
**Autonomy crafting action**			
ACACT → AUTSAT → ENGAGE	0.41	0.06	[0.29, 0.52]
ACACT → AUTSAT → TURN	−0.33	0.05	[−0.43, −0.24]
ACACT → AUTSAT → EFFORT	0.06	0.03	[0.00, 0.13]
**Competence crafting awareness**			
CCA → COMPSAT → ENGAGE	0.54	0.08	[0.39, 0.69]
CCA → COMPSAT → TURN	−0.26	0.06	[−0.38, −0.15]
CCA → COMPSAT → EFFORT	0.27	0.06	[0.17, 0.39]
**Competence crafting action**			
CCACT → COMPSAT → ENGAGE	0.28	0.07	[0.15, 0.41]
CCACT → COMPSAT → TURN	−0.13	0.04	[−0.22, −0.06]
CCACT → COMPSAT → EFFORT	0.14	0.04	[0.07, 0.22]
**Relatedness crafting awareness**			
RCA → RELSAT → ENGAGE	0.31	0.06	[0.20, 0.42]
RCA → RELSAT → TURN	−0.25	0.05	[−0.36, −0.16]
RCA → RELSAT → EFFORT	0.13	0.04	[0.05, 0.21]
**Relatedness crafting action**			
RCACT → RELSAT → ENGAGE	0.10	0.04	[0.01, 0.19]
RCACT → RELSAT → TURN	−0.08	0.04	[−0.15, −0.01]
RCACT → RELSAT → EFFORT	0.04	0.02	[0.00, 0.09]

Notes. BootSE—bootstrapped standard error; BootCI—bootstrapped confidence interval; ACA—autonomy crafting awareness; AUTSAT—autonomy satisfaction; ENGAGE—work engagement; ACACT—autonomy crafting action; TURN—turnover intention; EFFORT—work effort; CCA—competence crafting awareness; COMPSAT—competence satisfaction; CCACT—competence crafting action; RCA—relatedness crafting awareness; RELSAT—relatedness satisfaction; RCACT—relatedness crafting action. Unstandardized betas are reported.

**Table 7:** Direct effect of need crafting on employee ‘outcomes’.

Direct Effect	Estimate	Boot95%CI
Autonomy crafting awareness		
ACA → ENGAGE	0.03	[−0.05, 0.11]
ACA → TURN	0.29 ***	[0.17, 0.41]
ACA → EFFORT	0.48 ***	[0.35, 0.61]
**Autonomy crafting action**		
ACACT → ENGAGE	−0.12 **	[−0.21, −0.04]
ACACT → TURN	−0.01	[−0.14, 0.12]
ACACT → EFFORT	0.13	[−0.01, 0.28]
**Competence crafting awareness**		
CCA → ENGAGE	−0.21 ***	[−0.35, −0.07]
CCA → TURN	0.49 ***	[0.31, 0.67]
CCA → EFFORT	0.29 ***	[0.13, 0.44]
**Competence crafting action**		
CCACT → ENGAGE	0.02	[−0.11, 0.15]
CCACT → TURN	−0.36 ***	[−0.53, −0.19]
CCACT → EFFORT	0.04	[−0.11, 0.18]
**Relatedness crafting awareness**		
RCA → ENGAGE	−0.19 *	[−0.37, −0.01]
RCA → TURN	0.01	[−0.18, 0.20]
RCA → EFFORT	0.45 ***	[0.26, 0.63]
**Relatedness crafting action**		
RCACT → ENGAGE	0.25 **	[0.08, 0.42]
RCACT → TURN	0.06	[−0.11, 0.24]
RCACT → EFFORT	−0.08	[−0.25, 0.09]

Notes. BootCI—bootstrapped confidence interval; ACA—autonomy crafting awareness; AUTSAT—autonomy satisfaction; ENGAGE—work engagement; ACACT—autonomy crafting action; TURN—turnover intention; EFFORT—work effort; CCA—competence crafting awareness; COMPSAT—competence satisfaction; CCACT—competence crafting action; RCA—relatedness crafting awareness; RELSAT—relatedness satisfaction; RCACT—relatedness crafting action. *** *p* ≤ 0.001; ** *p* ≤ 0.01; and * *p* ≤ 0.05. Unstandardized betas are reported.

## Data Availability

The datasets generated and analyzed during the current study are not publicly available due to privacy concerns. However, they are available from the corresponding author on reasonable request.

## Supplementary Materials

The following supporting information can be downloaded at: https://www.mdpi.com/article/10.3390/bs14040301/s1, Figure S1: Simplified Representations of Specified Measurement Models (Models 1b, 3, and 4). Figure S2: Indirect Effect of Autonomy Crafting Awareness on Work Engagement through Autonomy Satisfaction. Figure S3: Indirect Effect of Autonomy Crafting Action on Work Engagement through Autonomy Satisfaction. Figure S4: Indirect Effect of Competence Crafting Awareness on Work Engagement through Competence Satisfaction. Figure S5: Indirect Effect of Competence Crafting Action on Work Engagement through Competence Satisfaction. Figure S6: Indirect Effect of Relatedness Crafting Awareness on Work Engagement through Relatedness Satisfaction. Figure S7: Indirect Effect of Relatedness Crafting Action on Work Engagement through Relatedness Satisfaction. Figure S8: Indirect Effect of Autonomy Crafting Awareness on Turnover Intention through Autonomy Satisfaction. Figure S9: Indirect Effect of Autonomy Crafting Action on Turnover Intention through Autonomy Satisfaction. Figure S10: Indirect Effect of Competence Crafting Awareness on Turnover Intention through Competence Satisfaction. Figure S11: Indirect Effect of Competence Crafting Action on Turnover Intention through Competence Satisfaction. Figure S12: Indirect Effect of Relatedness Crafting Awareness on Turnover Intention through Relatedness Satisfaction. Figure S13: Indirect Effect of Relatedness Crafting Action on Turnover Intention through Relatedness Satisfaction. Figure S14: Indirect Effect of Autonomy Crafting Awareness on Work Effort through Autonomy Satisfaction. Figure S15: Indirect Effect of Autonomy Crafting Action on Work Effort through Autonomy Satisfaction. Figure S16: Indirect Effect of Competence Crafting Action on Work Effort through Competence Satisfaction. Figure S17: Indirect Effect of Competence Crafting Action on Work Effort through Competence Satisfaction. Figure S18: Indirect Effect of Relatedness Crafting Awareness on Work Effort through Relatedness Satisfaction. Figure S19: Indirect Effect of Relatedness Crafting Action on Work Effort through Relatedness Satisfaction.
