# Supplementary material for: Evaluating Need Crafting: Scale Validation and Workplace Implications
Source: Behav Sci (Basel). 2024 Apr 5;14(4):301. doi: 10.3390/bs14040301 (PMC11047519; doi:10.3390/bs14040301)
Supplement: Supplementary file 1 [file behavsci-14-00301-s001.zip › behavsci-2908611-supplementary.pdf]

Figure S1. *Simplified Representations of Specified Measurement Models (Models 1b, 3, and 4)*

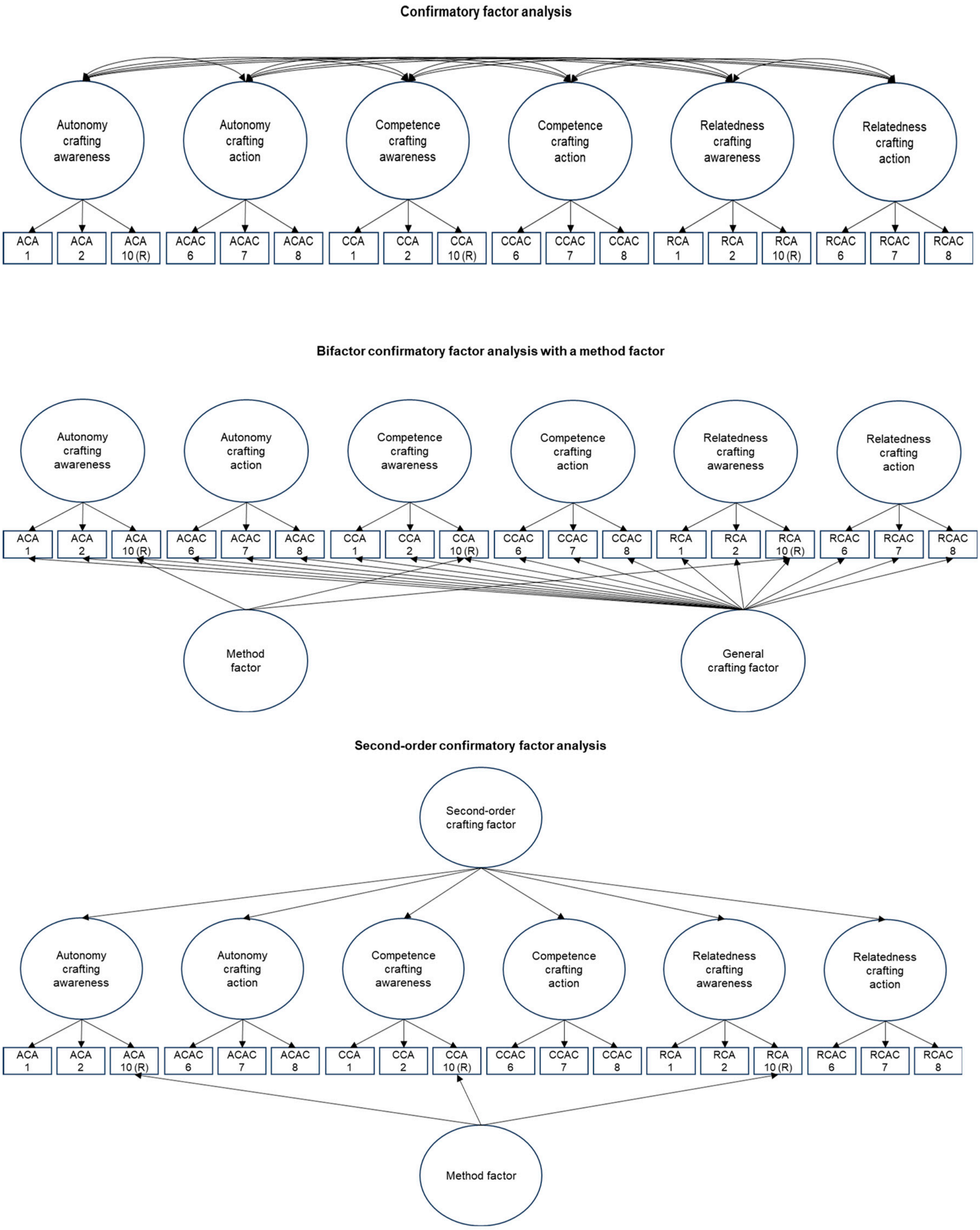

Figure S2

Indirect Effect of Autonomy Crafting Awareness on Work Engagement through Autonomy Satisfaction

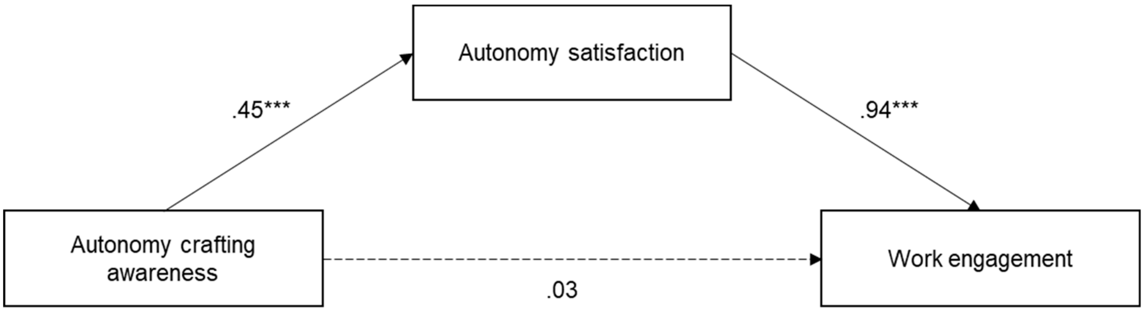

Notes. ***p < .001; dashed line indicates a non-significant association; for simplification, the covariate (autonomy crafting action) was omitted from the figure.

Figure S3

Indirect Effect of Autonomy Crafting Action on Work Engagement through Autonomy Satisfaction

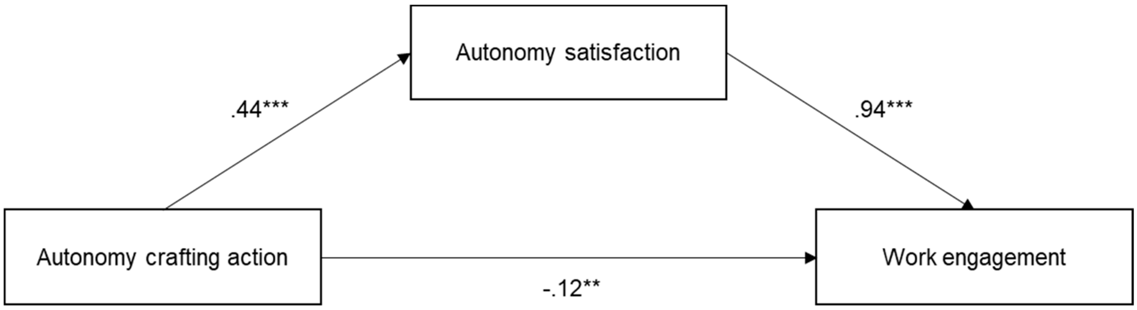

Notes. ***p < .001, **p < .01; for simplification, the covariate (autonomy crafting awareness) was omitted from the figure.

Figure S4

Indirect Effect of Competence Crafting Awareness on Work Engagement through Competence Satisfaction

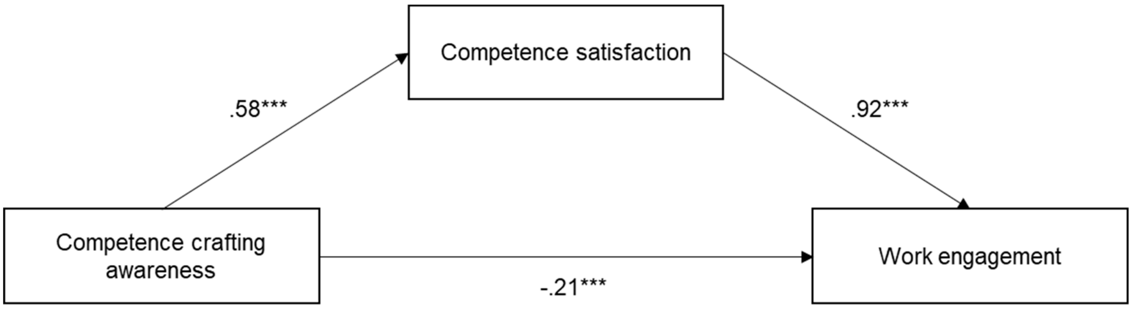

Notes. ***p < .001; for simplification, the covariate (competence crafting action) was omitted from the figure.

Figure S5

Indirect Effect of Competence Crafting Action on Work Engagement through Competence Satisfaction

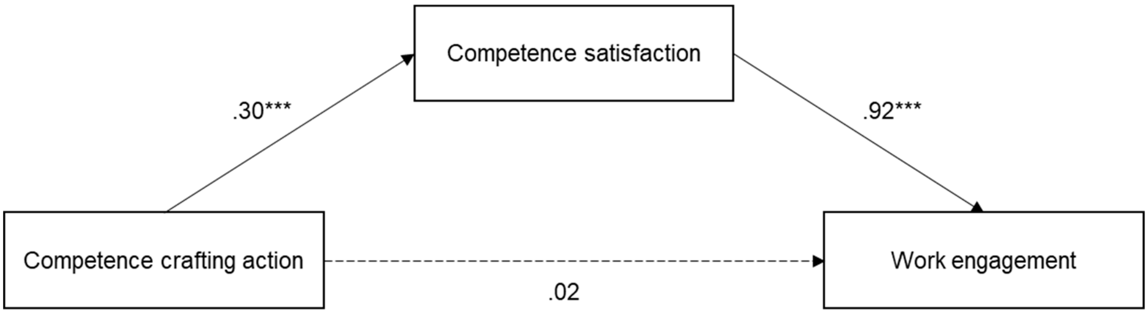

Notes. ***p < .001; dashed line indicates a non-significant association; for simplification, the covariate (competence crafting awareness) was omitted from the figure.

Figure S6

Indirect Effect of Relatedness Crafting Awareness on Work Engagement through Relatedness Satisfaction

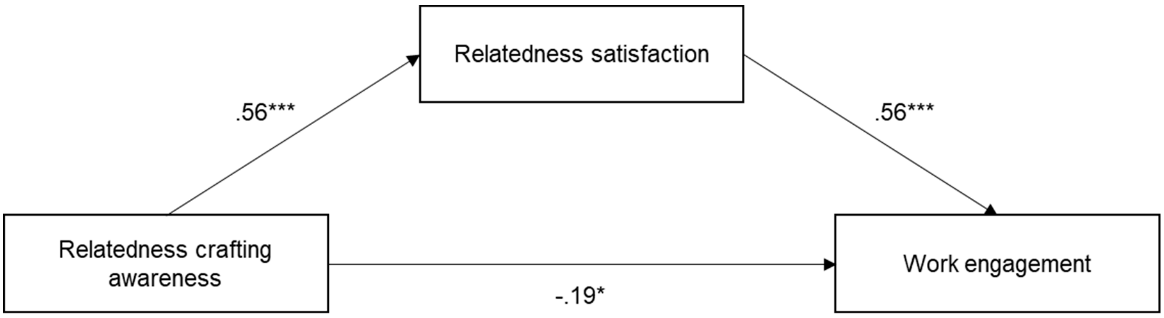

Notes. ***$p < .001$, $p < .05$; for simplification, the covariate (relatedness crafting action) was omitted from the figure.

Figure S7

Indirect Effect of Relatedness Crafting Action on Work Engagement through Relatedness Satisfaction

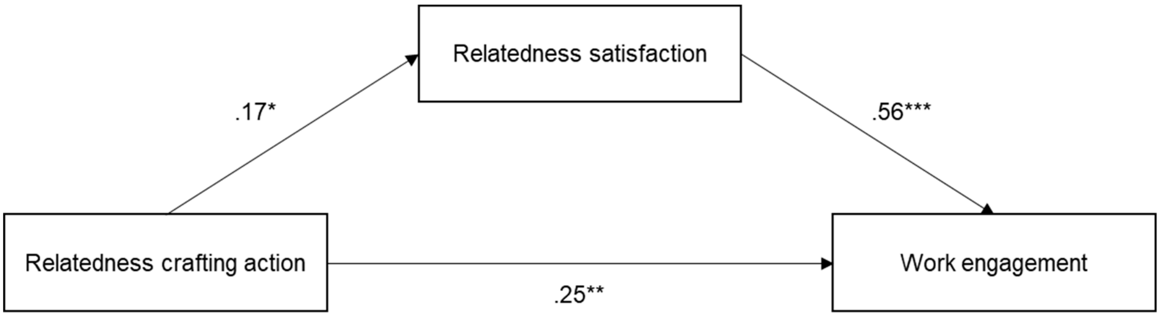

Notes. ***$p < .001$, $p < .01$, $p < .05$; for simplification, the covariate (relatedness crafting awareness) was omitted from the figure.

Figure S8

Indirect Effect of Autonomy Crafting Awareness on Turnover Intention through Autonomy Satisfaction

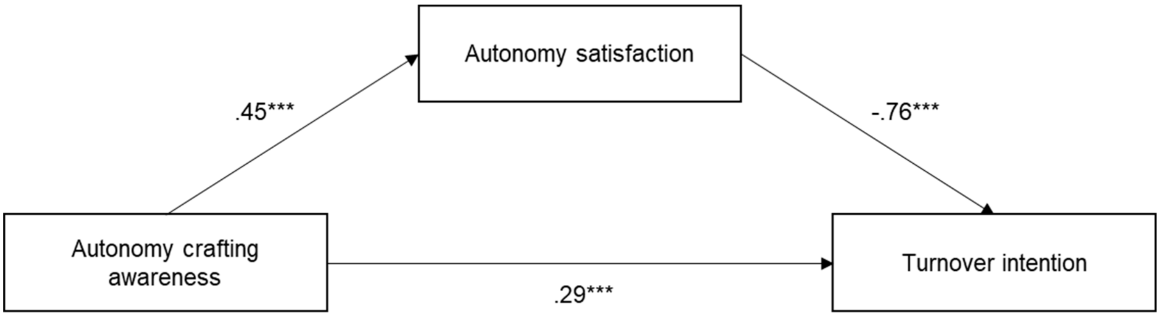

Notes. ****p* < .001; for simplification, the covariate (autonomy crafting action) was omitted from the figure.

Figure S9

Indirect Effect of Autonomy Crafting Action on Turnover Intention through Autonomy Satisfaction

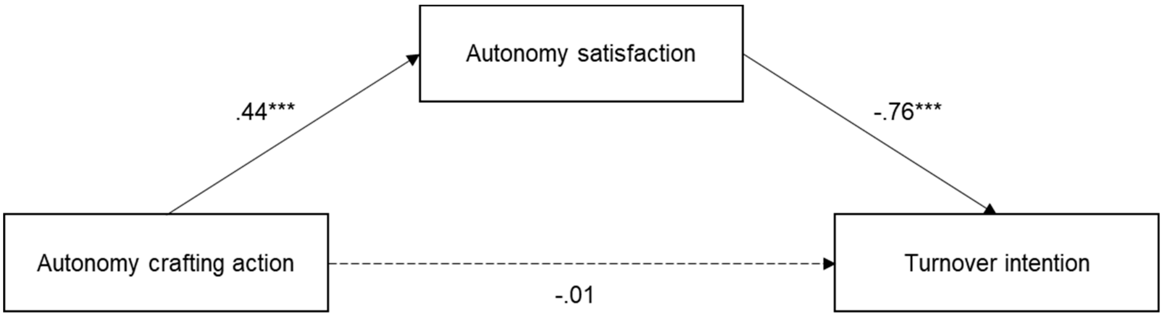

Notes. ****p* < .001; dashed line indicates a non-significant association; for simplification, the covariate (autonomy crafting awareness) was omitted from the figure.

Figure S10

Indirect Effect of Competence Crafting Awareness on Turnover Intention through Competence Satisfaction

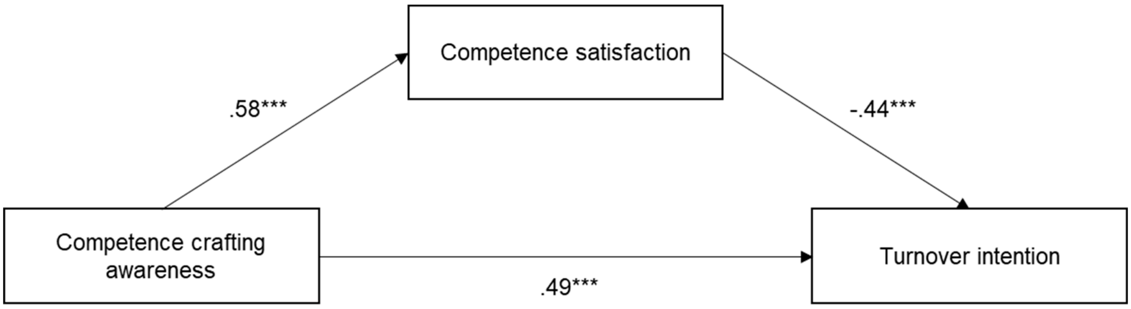

Notes. ****p* < .001; for simplification, the covariate (competence crafting action) was omitted from the figure.

Figure S11

Indirect Effect of Competence Crafting Action on Turnover Intention through Competence Satisfaction

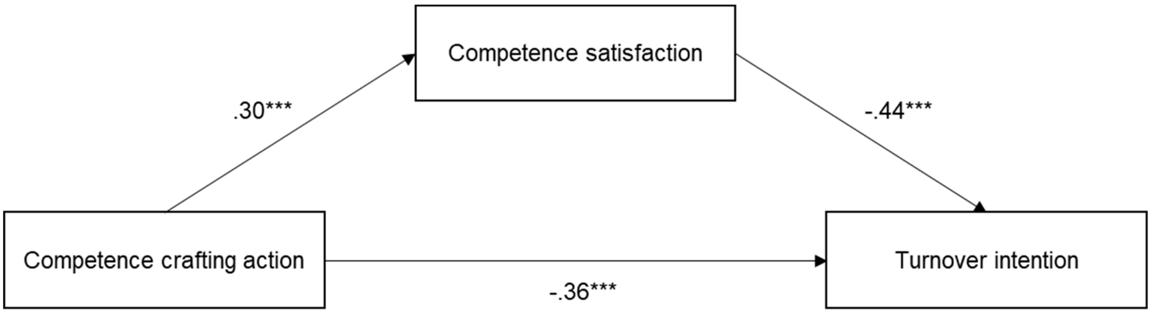

Notes. ****p* < .001; for simplification, the covariate (competence crafting awareness) was omitted from the figure.

Figure S12

Indirect Effect of Relatedness Crafting Awareness on Turnover Intention through Relatedness Satisfaction

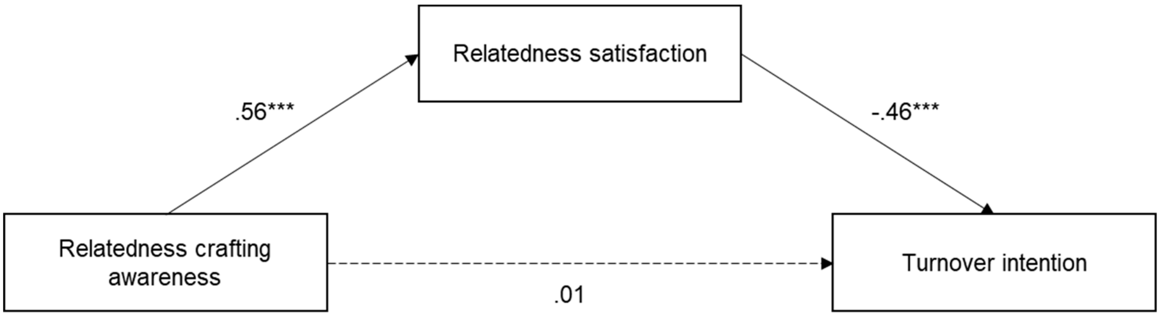

Notes. ****p* < .001; dashed line indicates a non-significant association; for simplification, the covariate (relatedness crafting action) was omitted from the figure.

Figure S13

Indirect Effect of Relatedness Crafting Action on Turnover Intention through Relatedness Satisfaction

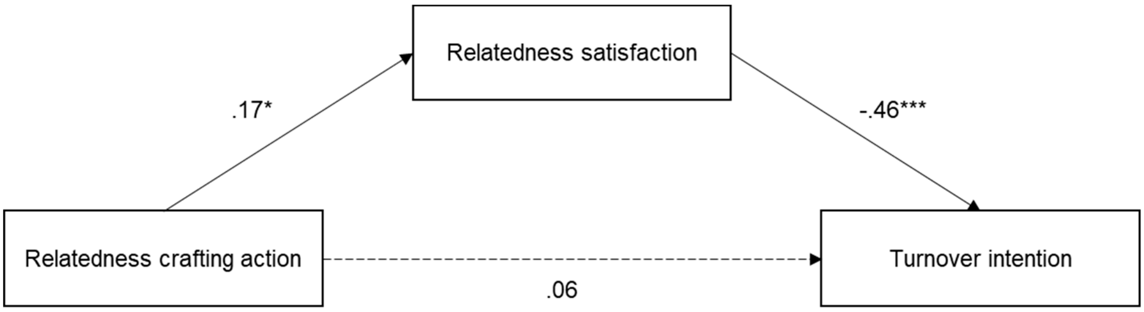

Notes. ****p* < .001, **p* < .05; dashed line indicates a non-significant association; for simplification, the covariate (relatedness crafting awareness) was omitted from the figure.

Figure S14

Indirect Effect of Autonomy Crafting Awareness on Work Effort through Autonomy Satisfaction

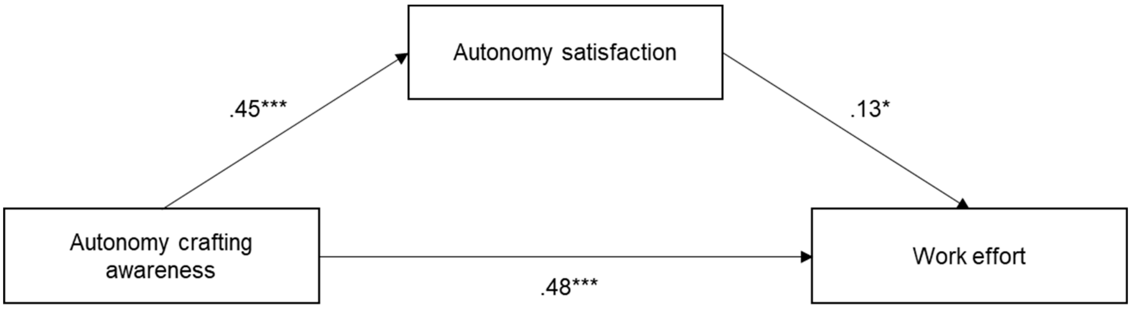

Notes. ***p < .001, *p < .05; for simplification, the covariate (autonomy crafting action) was omitted from the figure.

Figure S15

Indirect Effect of Autonomy Crafting Action on Work Effort through Autonomy Satisfaction

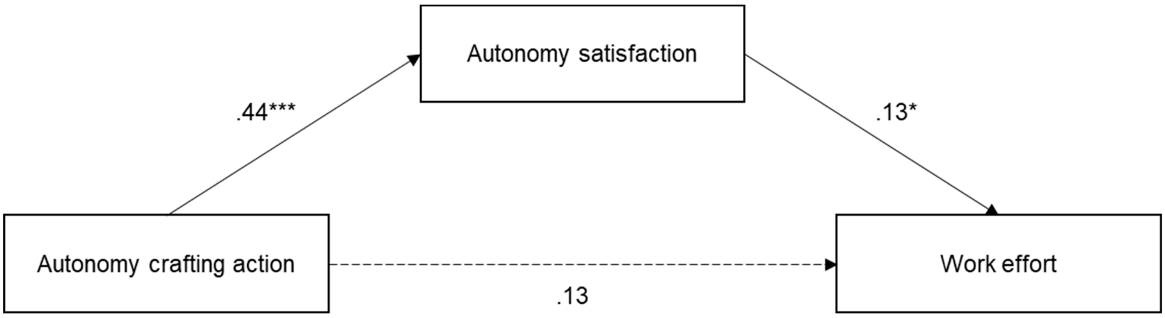

Notes. ***p < .001, *p < .05; dashed line indicates a non-significant association; for simplification, the covariate (autonomy crafting awareness) was omitted from the figure.

Figure S16

Indirect Effect of Competence Crafting Action on Work Effort through Competence Satisfaction

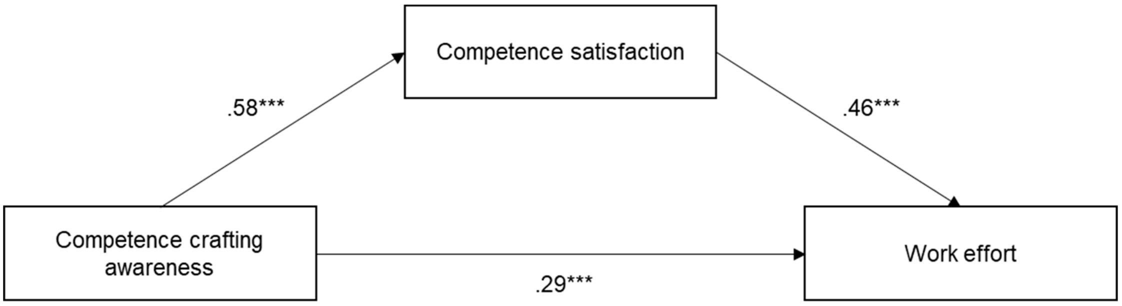

Notes. ****p* < .001; for simplification, the covariate (competence crafting action) was omitted from the figure.

Figure S17

Indirect Effect of Competence Crafting Action on Work Effort through Competence Satisfaction

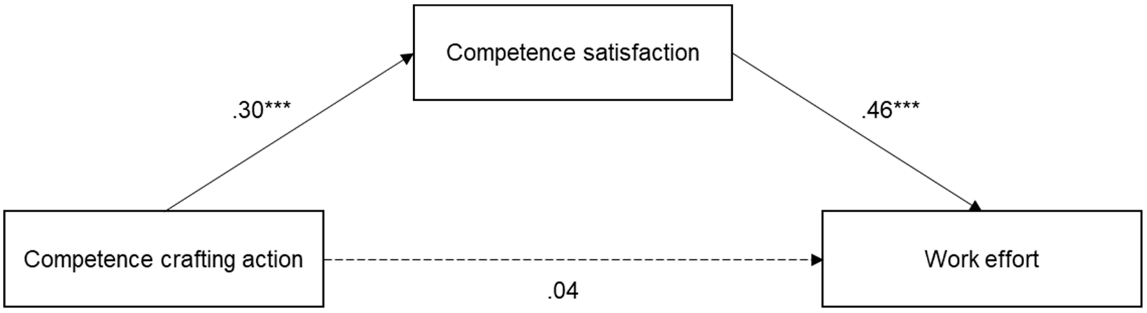

Notes. ****p* < .001; dashed line indicates a non-significant association; for simplification, the covariate (competence crafting awareness) was omitted from the figure.

Figure S18

Indirect Effect of Relatedness Crafting Awareness on Work Effort through Relatedness Satisfaction

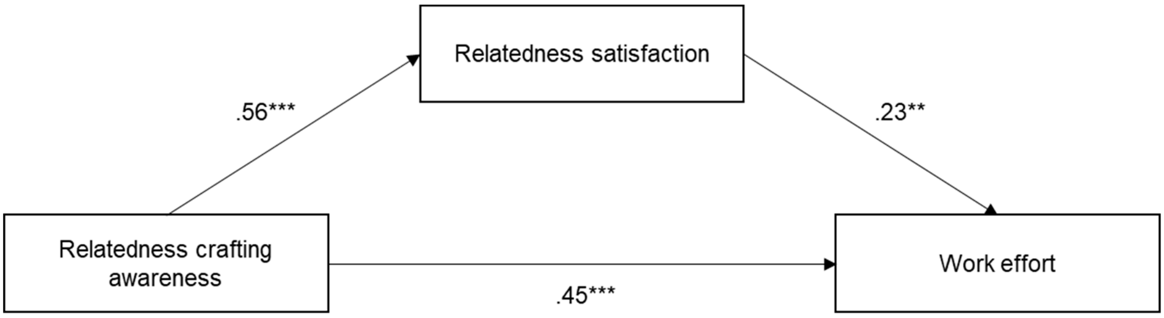

Notes. ***p < .001; for simplification, the covariate (relatedness crafting action) was omitted from the figure.

Figure S19

Indirect Effect of Relatedness Crafting Action on Work Effort through Relatedness Satisfaction

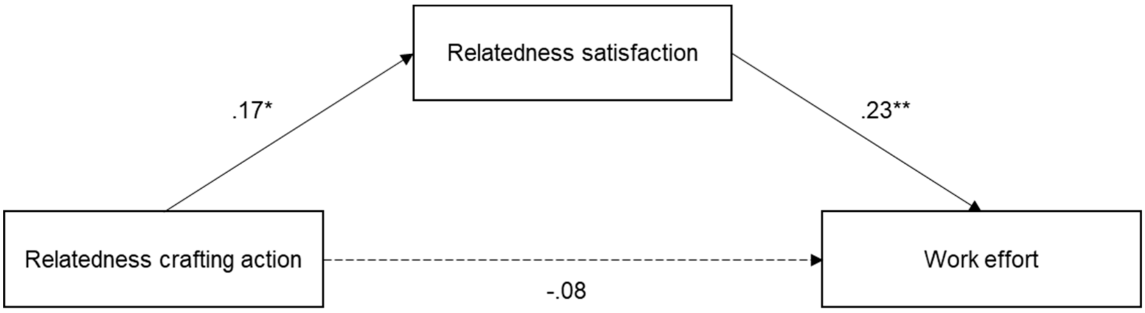

Notes. ***p < .001, p < .05; dashed line indicates a non-significant association; for simplification, the covariate (relatedness crafting awareness) was omitted from the figure.
